# Solution Behavior and Activity of a Halophilic Esterase under High Salt Concentration

**DOI:** 10.1371/journal.pone.0006980

**Published:** 2009-09-14

**Authors:** Lang Rao, Xiubo Zhao, Fang Pan, Yin Li, Yanfen Xue, Yanhe Ma, Jian R. Lu

**Affiliations:** 1 Institute of Microbiology, Chinese Academy of Sciences, Beijing, China; 2 Biological Physics Laboratory, School of Physics and Astronomy, the University of Manchester, Manchester, United Kingdom; University of Strathclyde, United Kingdom

## Abstract

**Background:**

Halophiles are extremophiles that thrive in environments with very high concentrations of salt. Although the salt reliance and physiology of these extremophiles have been widely investigated, the molecular working mechanisms of their enzymes under salty conditions have been little explored.

**Methodology/Principal Findings:**

A halophilic esterolytic enzyme LipC derived from archeaon *Haloarcula marismortui* was overexpressed from *Escherichia coli* BL21. The purified enzyme showed a range of hydrolytic activity towards the substrates of *p*-nitrophenyl esters with different alkyl chains (n = 2−16), with the highest activity being observed for *p*-nitrophenyl acetate, consistent with the basic character of an esterase. The optimal esterase activities were found to be at pH 9.5 and [NaCl] = 3.4 M or [KCl] = 3.0 M and at around 45°C. Interestingly, the hydrolysis activity showed a clear reversibility against changes in salt concentration. At the ambient temperature of 22°C, enzyme systems working under the optimal salt concentrations were very stable against time. Increase in temperature increased the activity but reduced its stability. Circular dichroism (CD), dynamic light scattering (DLS) and small angle neutron scattering (SANS) were deployed to determine the physical states of LipC in solution. As the salt concentration increased, DLS revealed substantial increase in aggregate sizes, but CD measurements revealed the maximal retention of the α-helical structure at the salt concentration matching the optimal activity. These observations were supported by SANS analysis that revealed the highest proportion of unimers and dimers around the optimal salt concentration, although the coexistent larger aggregates showed a trend of increasing size with salt concentration, consistent with the DLS data.

**Conclusions/Significance:**

The solution α-helical structure and activity relation also matched the highest proportion of enzyme unimers and dimers. Given that all the solutions studied were structurally inhomogeneous, it is important for future work to understand how the LipC's solution aggregation affected its activity.

## Introduction

Lipolytic enzymes (lipases and esterases) primarily catalyze both the hydrolysis and synthesis of ester compounds and the catalytic processes are widespread in various organisms including animals, plants, and microorganisms. Both types of enzymes belong to the serine hydrolase family and share the structural and functional characteristics, including a catalytic triad Gly-X-Ser-X-Gly, an α/β hydrolase fold and a cofactor independent activity [1], [2]. Interfacial activation and the presence of a surface loop covering the activity site are the structural hallmark that distinguishes lipases from esterases [3], [4]. Because of their broad substrate specificity, highly chemo-, regio-, enantio-selectivity and non-aqueous catalytic properties, lipases and esterases have many applications in biotechnology, ranging from their use as addictives in laundry detergents and textiles to stereo-specific biocatalysis and pharmaceutical production [5], [6]. To date, lipolytic enzymes like esterases and lipases represent one of the largest groups of industrial enzymes accounting for billions of dollars every year [7]. Industrial applications often require aggressive reaction conditions like high salt contents, high/low temperature, and low water activity. However, normal hydrolytic catalysts usually can't meet these requirements. Thus, novel hydrolytic catalysts with better catalytic efficiency and specific properties suitable for special reaction conditions are in high demand.

Extremophiles dwelling in abnormal environments (high/low temperature, extreme pH, and salinity) are good candidates for selecting novel lipolytic enzymes [8], [9]. So far, most extensively studied extreme lipases and esterases are from thermophilic [10], [11], [12], [13], [14], alkaline [15], [16], psychrophilic [17], [18], [19] and halophilic environments [20], [21], [22], [23]. Halobacteria living in high saline ecosystems can have optimal salt concentrations for growth that amount to some ten times above the sea water. Their enzymes have developed particular features including adequate stability and solubility in high salt concentrations by acquiring a relatively large number of charged amino acid residues on their surfaces to prevent precipitation [24], [25]. As high salt concentration tends to greatly reduce the water activity of the medium it is reasonable to apply this kind of enzymes in solvent tolerant process [9].

Since Norberg and von Hofsten's pioneering research on hydrolytic enzymes from halophilic organisms by demonstrating the presence of extracellular proteases from halophilic bacteria, an extensive list of amylases, nucleases, xylosidases, lipases and esterases purified from halophilic organisms has been reported [26]. Gonzalez and Gutierrez also reported the extraction of lipolytic enzymes from halophiles that showed the presence of surfactant (Tween 20–80) degrading activities among strains of halobacteria [20]. After this period of the early work, there were subsequently little research activities on halophilic lipases. In 2005, two halobacterial secreted lipases were isolated which could hydrolyse both butyrate and olive oil [27]. This was the first report of the halophilic lipases that showed hyper saline tolerance and good thermo-stability [22]. Sana et al purified an esterase with its optimal activity at 10% NaCl. The same esterase also showed an extreme stability in 50% DMSO. This example indicated that it was possible to explore organic tolerant lipolytic enzymes from saline tolerant bacteria [28]. Archaeon *Haloarcula marismortui* (ATCC 43049) is a typical halophilic microorganism which was found in the Dead Sea and grows at the high NaCl concentration around 4 M [21]. With its genomic sequence information [29], [30], a putative lipase/esterase gene lipC with an α/β hydrolase fold was discovered.

As the halophilic esterase is a truly salt dependent esterase, it is a good model for studying the effects of salt concentration and temperature on its activity and the structural implication in aqueous solution. In this work, we present the cloning, heterologous expression of the lipolytic gene lipC from halophilic Archaeon *Haloarcula marismortui* (ATCC 43049) using mesophilic host *Escherichia coli* BL21 (DE3). The heterologous enzyme was purified and their enzymatic properties were studied. In order to illustrate relationship between the physical state of the enzyme in solution and its activity, the catalytic behaviour of the enzyme and its different structural properties under different solution environments were studied by combining bioactivity assays with dynamic light scattering (DLS) and small angle neutron scattering (SANS). Our studies have revealed that the halophilic enzyme displayed hydrolytic activity over an alkaline pH range. At pH 9.5, its optimal activities occurred at the temperature of 45°C and a high NaCl concentration of 3.4 M. Shift of NaCl concentration away from the optimal value led to the decline of enzymatic activity, but readjustment of salt concentration back to the optimal condition could restore the optimal activity. Structural studies from SANS revealed that under the optimal activity conditions the enzyme existed mostly in the form of free molecules. Decrease in salt concentration improved the solubilisation of the enzyme whilst increase in salt concentration above the optimal concentration intensified aggregation. Interestingly, however, the internal secondary structures as revealed by circular dichroism (CD) measurements became maximal around the optimal solution sat concentration range. As the salt concentration was shifted away from the optimal conditions substantial loss of secondary structures occurred, consistent with the trend of activity. These results thus demonstrated a clear correlation between enzymatic activity and the secondary structure change, but the molecular mechanism of salt effect on the solubilization and aggregation of the enzyme requires further investigation.

## Results and Discussion

### 1. Analysis of nucleotide and amino acid sequence of halophilic esterase LipC

We undertook the blast analysis of our enzyme LipC in the hydrolysis database ESTHER (http://www.ensam.inra.fr/cgi-bin/ace/index) and the results showed that the LipC sequence belonged to the H block of the lipase/esterase family corresponding to the family IV of bacterial lipolytic enzymes according to Arpigny and Jaeger classification (1999) [31] and that the catalytic triad of LipC was Ser128, Asp224 and His254. The sequence of this enzyme only had some 35% matching to those of the existing ones searched from GenBank, consistent with the outcome of Müller-Santos et al [30]. Furthermore, the enzyme contained 17% acidic amino acids and had an isoelectric point of 4.2, showing that it was an unusual enzyme, as far as the primary sequences were concerned. Also, neither transmembrane domains nor signal peptides were identified, indicating that it was a cytosolic enzyme. The distribution of high negative surface charge groups may favor solvation, in a manner similar to the glucose dehydragenase from *Haloferax mediterranei*. The solvent exposed glutamate and aspartate residues are strongly hydrated and as a result, the degree of surface solvation of this glucose dehydrogenase is among the highest in proteins with known 3D structures. The association of these hydrated amino acid residues with cations would substantially contribute to the stability and solubility of LipC or alike halophilic enzymes in high salt or solvent deprived environments.

### 2. Cloning, expression and purification

**Figure 1:**
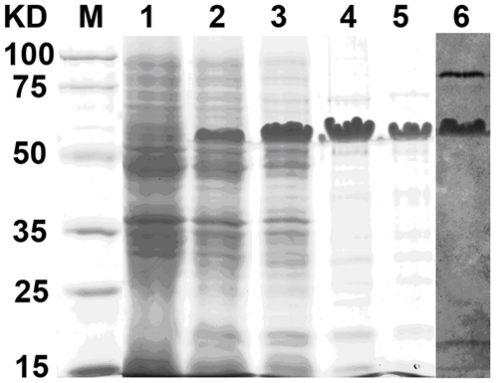
SDS-PAGE analysis of different samples taken during the purification process of esterase LipC. Lane 1: crude supernatant of pET28a-LipC non-induced culture; Lanes 2 and 6: crude extract of induced culture; Lane 3: purification on Ni-NTA affinity Sepharose column; Lane 4: further purification with DEAE column; Lanes 5 and 6: further purification with Sephadex-G200. Lanes 1 to 5 were stained with Coomassie Brilliant Blue and Lanes 6 was stained with α-naphtyl acetate and Fast Blue for the detection of hydrolase activity.

The esterase gene was amplified by PCR from the genomic DNA of *Haloarcula marismortui* (ATCC 43049). Recombinant esterase was expressed by pET28a in *E. coli* BL21 (DE3). SDS-PAGE analysis showed that esterase LipC was soluble upon binding with SDS (Lane 5, Figure 1) and was expressed as active enzyme after staining with α-naphtyl acetate and Fast Blue for the detection of hydrolase activity (Lane 6, Figure 1). The esterase activity staining was performed in non-denaturing PAGE analysis and the result confirmed that LipC performed its esterase activity as free monomers. Purification was achieved with Ni-NTA, DEAE chromatography and gel filtration chromatography and the yielding was summarised in Table 1. The molecular mass of LipC measured by SDS-PAGE analysis (Lanes 3–5, Figure 1) is approximately 50 kDa and is apparently heavier than the theoretical weight of 34 kDa as calculated from the amino acid sequence.

**Table 1:** Purification steps and yields of recombinant esterase from *Haloarcula marismortui* (ATCC43049).

	Crude extract	Ni-NTA column	DEAE column	Superdex 75 column
Total activity (mU)	57600	43000	33264	22170
Total protein (mg)	664	237	72	28
Specific activity (mU/mg)	85	181	462	792
Purification fold	1	2.1	5.4	9.3
Yield (%)	100	75.5	57.8	38.5

The activities were measured using 1 mM p-nitrophenyl acetate as a substrate.

**Figure 2:**
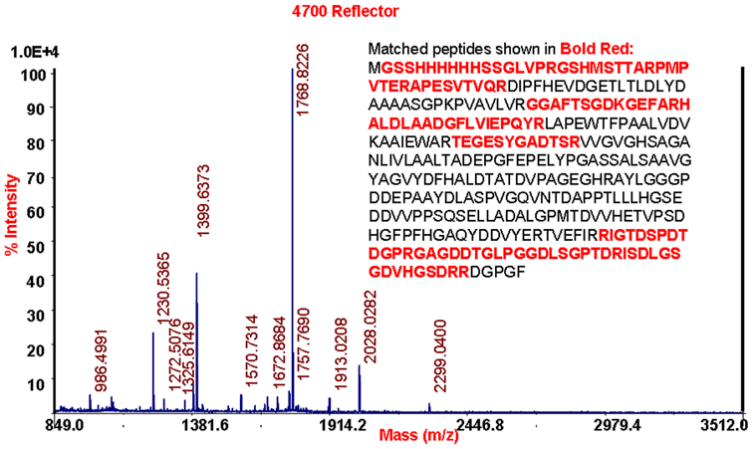
MALDI-TOF peptide mass fingerprint (PMF) spectrum. The PMF analysis was made from fragments of halophilic esterase LipC derived through trypsin digestion. The expected tryptic masses clearly matched, with 1 Da tolerance, the calculated values. The sequence coverage of these fragments is shown in red.

In order to further identify the expressed protein we applied PMF spectral analysis on the target protein of SDS-PAGE. The peptide mass fingerprint (PMF) spectrum (Figure 2) of fragments of LipC derived through trypsin digestion showed that the expressed protein had the amino acid sequence identical to the theoretical sequence, confirming that the protein was correctly expressed. It has been demonstrated that halophilic proteins bind less SDS than their non-halophilic counterparts, resulting in reduced mobility and overestimation of molecular weights [32]. The slow migration of LipC seemed to arise from the lack of SDS binding that could be deterred by the unusually high negative charge amino acids occupying about 17% of the total MW. Although the high content of negative charge amino acids was expected to help speed up the migration, the outcome suggested that SDS binding was still significant and played a dominant role.

### 3. Substrate specificity

**Figure 3:**
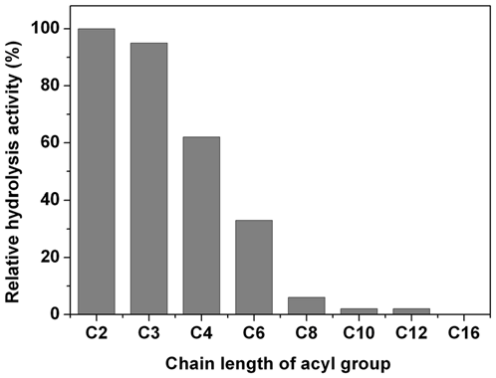
Substrate specificity of the purified esterase LipC. The enzyme activity was measured towards p-nitrophenyl acylates with different acyl chain lengths (C2–C16) at 45°C and pH 8.5 with 3.4 M NaCl present. Activity for p-NP-C2 was taken as 100%.

The rates of hydrolysis of p-nitrophenyl acylates with chain lengths between C2 and C16 were determined. The highest specific activity of LipC was found towards the short chain p-nitrophenyl esters of p-NP-C2 and p-NP-C3. With chain length increasing, the activity started to decrease and dropped to the baseline as evident from C10 onwards (Figure 3). No activity toward p-NP-C16 and olive oil was detected at all, confirming that LipC was an esterase rather than a lipase.

### 4. Effects of pH and temperature on lipase activity

**Figure 4:**
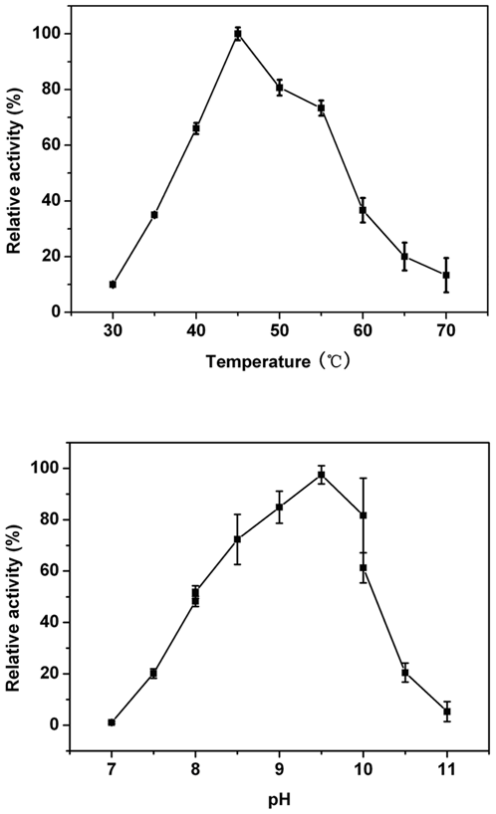
Influences of (A) temperature and (B) pH on the esterase activity of LipC of *Haloarcula marismortui* (ATCC 43049). For the pH profile, the activity was measured at constant ionic strength of 3.4 M NaCl and ambient temperature of 22 C. For the temperature profile, the activity was measured in 50 mM Tris-HCl and pH 8.5 with no NaCl added. The values represented the means of results from duplicate experiments and activities at the peak positions were taken as references.

The effect of temperature on LipC activity was first assessed in 50 mM Tris-HCl and pH 8.5, using p-NP-C4 as substrate. The most favourite substrate p-NP-C2 was not used because its hydrolysis under the optimal salt and pH ranges was exceedingly fast. The activity peaked at the temperature around 45°C. The results shown in Figure 4 (top) were plotted by taking the activity at 45°C as 100% (reference). It can be seen from Figure 4 that away from the optimal temperature the relative activity declined sharply, implying a sensitive response in the thermal stability of the enzyme. The effect of pH on esterase activity was also assessed at 45°C (Figure 4, bottom), again using p-NP-C4 as substrate. The enzyme was active in the pH range of 7.5–11.0, with its maximal activity at pH 9.5. These results were broadly consistent with the work by Müller-Santos et al.[30] and the differences in the exact optimal values in pH and temperature may reflect the use of different substrates and the combination of system conditions.

### 5. Effect of salinity on the activity

**Figure 5:**
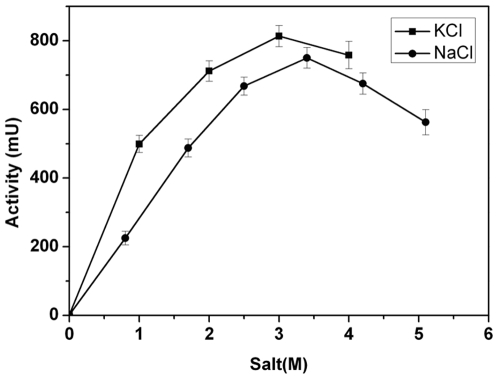
Effects of salt on *p*-NP-C2 hydrolysis activity of LipC. For each salt, the activity assessment was made at 45°C, pH 8.5 in Tris-HCl buffer, All the assays were taken when the enzyme incubated with the salt for more than 1 hour.

As LipC was of the halophilic origin, it is useful to examine how salt or ionic strength affected enzymatic activity. Figure 5 shows the hydrolysis activity of LipC responded sensitively to [NaCl] and [KCl]. When the enzyme was incubated in 3.4 M NaCl or 3 M KCl its activity reached the respective peak values. Shift in salt concentration away from the optimal values caused decline in enzyme activity. At very low or very high salt concentrations, little hydrolysis activity could be detected, but it was found that enzyme activity could be gradually restored when salt concentration was adjusted close to the optimal salt concentration range, followed by incubation of the solution for 3–5 hours or more. The reversible activity changes appear to be a very characteristic feature of this halophilic enzyme.

### 6. Stability of LipC

**Figure 6:**
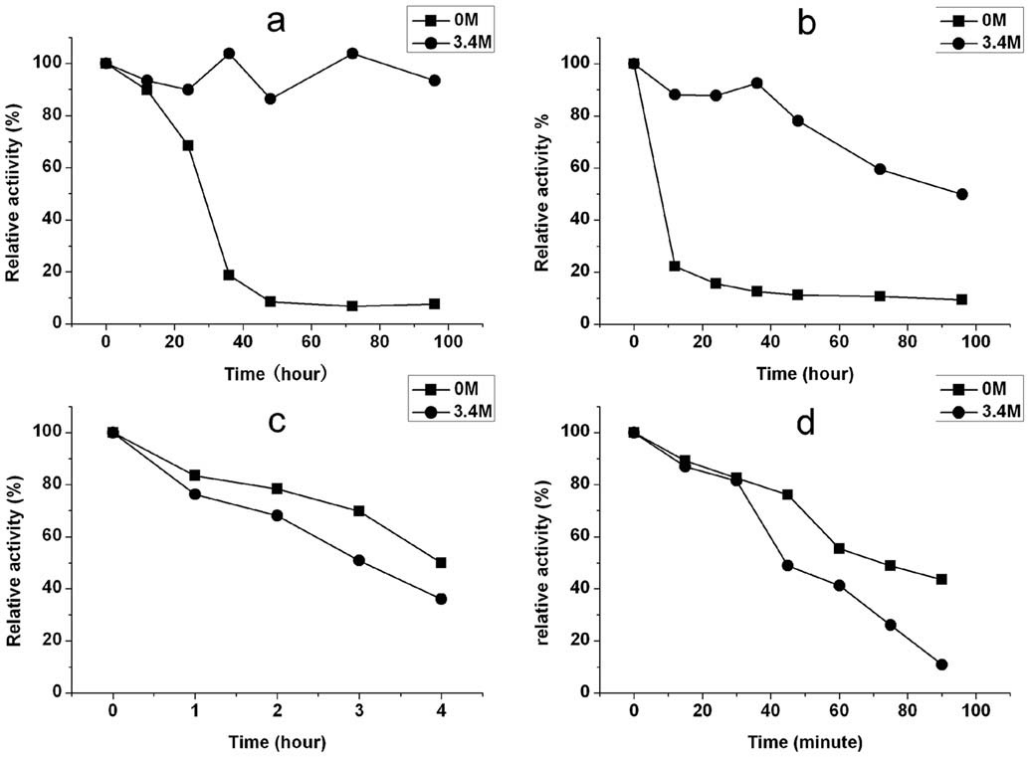
Effects of temperature and [NaCl] on the stability of LipC. Measurements were made at 1 mg/ml enzyme with (•) and without (▪) 3.4 M NaCl: (a) 22°C, (b) 37°C, (c) 55°C and (d) 60°C. Relative activities (normalised to the value at the start of the measurements) were determined at 50 mM Tris-HCl, pH 8.0 using *p*-NP-C2 as substrate.

The above studies have assessed the changes of activity with respect to temperature, pH and ionic strength and a typical combination of the optimal conditions for achieving the highest enzyme activity is at pH 8.5, 45 C and [NaCl] = 3.4 M. However, enzymatic activity often decays against time. In addition, it is useful to examine the interplay between these key factors. Thus we have interrogated the stability of LipC under a set of combined conditions with the results shown in Figure 6. In each case, the relative activity was screened against time. The results show that high salt concentrations can stabilise the halophilic protein in the aqueous solution, an observation consistent with the report by Boutaiba et al. [22].

However, the influence of NaCl on the stability of LipC is highly temperature dependent. It can be seen from Figure 6 that at the ambient temperature of 22***°***C, the enzyme retained its highest activity when incubated at the high salt concentration of 3.4 M NaCl, for well over 100 hours (the period during which the observation was conducted). In contrast, the enzyme steadily lost its activity without salt, with most of its activity being lost within 50 hours. However, as the incubation temperature was raised to 37***°***C, the activities under both solution conditions showed a clear sign of decline, although the presence of 3.4 M NaCl sustained the activity more effectively. When the incubation temperature reached 55°C, the rates of activity loss were significantly increased with and without the addition of 3.4 M NaCl, but the enzyme in the high salt solution denatured faster, showing that addition of salt accelerated activity loss. With further increase in temperature to 60***°***C the trend of acceleration of activity loss continued and salt addition again accelerated the rate of denaturation, confirming that whilst salt addition helped stabilize the enzyme below 50°C, the opposite effect was produced above it.

### 7. CD Spectra

**Figure 7:**
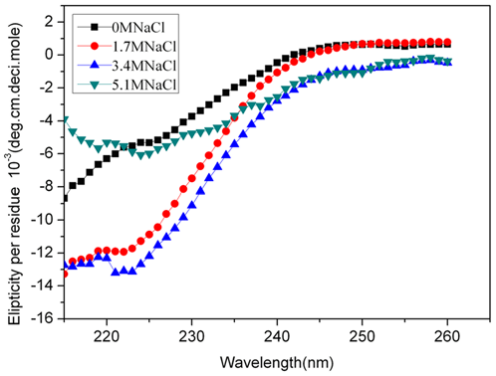
Far-UV CD spectra of LipC. The CD measurements were made in the presence of different concentrations of NaCl: 0 M (▪), 1.7 M (•), 3.4 M (▴) and 5.1 M (▾). The concentration of LipC was fixed at 0.4 mg/ml (22°C, 20 mM Tris-HCl buffer pH 8).

The CD spectra of LipC, measured at different NaCl concentrations with the temperature kept constant at 22***°***C and pH at 8, are shown in Figure 7. All samples were incubated for about 1 hour for equilibration before the CD measurements were made. The results show obvious changes in the peak positions of negative ellipticity at 222–225 nm, characteristic of the spectral profile of the α-helical structure [33]. When the NaCl concentration increased from zero to 1.7 M, there was a large increase of the negative ellipiticity peak at 222–225 nm, but only minor change was observed when salt concentration was raised from 1.7 M to 3.4 M. However, when the salt concentration further increased to 5.1 M, there was an obvious decrease of the 222 nm signal intensity. Compared with the CD spectra obtained at 1.7 M and 3.4 M, the spectrum of LipC at the highest salt concentration was more like that without salt added. Thus the CD spectra have unraveled changes in the secondary structure and folding properties of the protein against the variation of [NaCl] in aqueous solution. The CD results indicate that the enzyme adopted an inadequate secondary structural conformation at low and high salt concentrations as studied and could not perform its catalytic activity. With the addition of an intermediate and yet adequate [NaCl], the enzyme adopted its proper conformation as marked by the formation of the α-helix dominant structure, which in turn triggered its catalytic ability. The enzyme reached its peak catalytic ability at 3.4 M NaCl solution when its conformation adjusted to the most characteristic α-helix dominant structure.

### 8. DLS measurements

**Figure 8:**
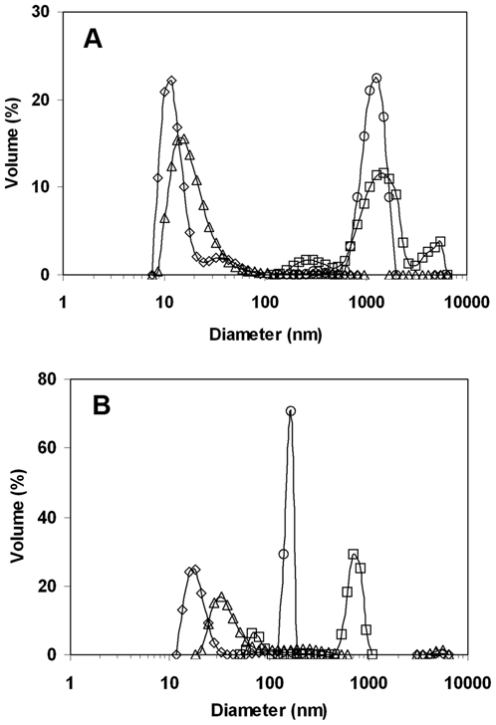
Dynamic light scattering from different LipC solutions. The DLS measurements were made from the esterase in aqueous buffer (20 mM TrisHCl, pH 8) at 22°C (◊), 45°C(Δ), and buffer with 3.4 M NaCl at 22°C (□), 45°C (○). The enzyme concentrations were between 0.5 and 0.7 mg/ml. A, measured immediately; B, measured after 2 days.

The physical state of LipC in aqueous solution under different conditions was subsequently investigated by DLS with representative data shown in Figure 8. In DLS experiments, particles in solution are illuminated with light of a given wavelength (λ = 632.8 nm used in this work) and the intensity fluctuations from the scattered light are measured over a time course typically between 1 ns and 1 ms. The intensity fluctuations occur due to the random diffusion of particles through the solvent, known as Brownian motion. The change in signal intensity over the time course of an experiment arising from these fluctuations can be principally described through the autocorrelation function. The significance of the autocorrelation function is that particles with a larger hydrodynamic radius will diffuse with a lower velocity through the bulk solvent. As a result, the autocorrelation function, describing the similarity between the two signals, will be greater for particles with a larger hydrodynamic radius, *i.e.*, the signal from a larger particle will not have altered as much over time as that from a smaller particle. If the particles studied are small compared to the employed wavelength, the translational diffusion coefficient, *D_o_*, can be determined through the Laplace inversion of the autocorrelation function. From the diffusion coefficient, the hydrodynamic radius of the scattering particles can be calculated from the Stokes-Einstein equation [34]:
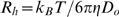
(1)where *k_B_* denotes the Boltzmann constant, *T* the absolute temperature, and *η* is the viscosity of the solvent in the same temperature. *R_h_* is experimentally the measure of the maximal radius of a hydrated object (assuming it rotates along all directions). In the case of a non-spherical object, it approximates to the largest rotational radius. DLS cannot provide information about the shape of a particle.

The main size distribution of the sample in 20 mM Tris-HCl buffer at 22***°***C and pH 8 was found to peak around 10 nm, with a shoulder peak centred around 30 nm. Globular proteins such as lysozyme (MW = 14 kD), hCG (human chorionic gonadotrophin, MW = 38 kD) and BSA (MW = 67 kD) are ellipsoidal or cylindrical in shape and have a length of *ca* 4.5 nm, 7 nm and 14 nm, respectively. Given that LipC had similar MW as hCG, the main size distribution centered around 10 nm could be a measure of the hydrodynamic diameters of the molecules under the solution environment. Assuming that the main peak dimension reflected the length of LipC, the result would indicate that the majority of LipC molecules could exist as individually dispersed molecules, with a small proportion of them present in the form of small aggregates. The effect of temperature on the state of aggregation was studied by raising the pH 8 enzyme solution (without any NaCl added) from 22°C to 45°C, with the size distribution similarly measured by DLS. As can be seen from Figure 8, the overall distribution closely resembled that at the ambient temperature, but the peak has shifted to a greater value of ca 15 nm, indicating some modest increase of the size of the average scattering objects. In addition, the shoulder peak has disappeared, but the distribution is broader and is skewed towards the large size side, showing the possible coexistence of relatively large aggregates with unimers and dimers. These DLS data have thus revealed that whilst LipC molecules were predominantly dissolved under these conditions some extent of aggregation occurred and that the solutions were inhomogeneous.

The effect of salt addition on enzyme solubilization and aggregation was examined by undertaking the above DLS measurements in the presence of 3.4 M NaCl. The main observation from Figure 8 is that salt addition has substantially increased the sizes of the scattering objects, evident from the shift of peaks to much greater values. At both temperatures, the main peaks are centered around 1000 nm (consistent with the occurrence of some turbidity), indicating the formation of large aggregates in these systems. Furthermore, the size distribution measured at the ambient temperature is characterized by 3 peaks. The convergence to the single, middle, peak at the high temperature suggests that temperature increase in the presence of 3.4 M NaCl has made the aggregates more uniform.

The time dependent change of solution aggregation has also been assessed and the results are shown in Figure 8 as well. After 2 days of equilibration, the two smaller size distributions were shifted to slightly bigger sizes, but the two large size distributions in the presence of 3.4 M NaCl were shifted to smaller size ranges. The largest drop in size was observed from enzyme dissolved in 3.4 M NaCl at 45°C, with the peak aggregate diameter decreased from some 1000 nm to about 200 nm only. A further observation was that all four distributions become more uniform and symmetrical. But the main outcome from the DLS studies was the formation of large enzyme aggregates upon addition of 3.4 M NaCl where the enzyme was rather active.

### 9. SANS

SANS is a well established technique for investigating the size distributions of surfactant and polymer micelles and other nanoparticles in aqueous solution. In comparison with DLS, it has greater resolution and sensitivity and is capable of detecting the shape of nanoparticles as well. SANS is widely used for characterizing the size and shape of protein molecules under different conditions. Size increase with reference to the crystalline structure of the enzyme is usually attributed to solvation or molecular aggregation driven by structural deformation and unfolding.[34], [35]

SANS measures the differential scattering cross-section, (d*Σ*/d*Ω*)(*Q*), which contains information on the size, shape and interactions between the scattering centers or particles in the sample [36]. A generalized expression for the small angle neutron scattering from any sample is given in equation (2):
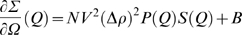
(2)where *N* is the number concentration of scattering centers, *V* (or *V_p_*) is the volume of one scattering centre, Δ*ρ* is the contrast, indicating the difference in scattering length density between the scattering object and the surrounding solvent and *B* is the background signal. *S*(*Q*) is the interparticle form factor and *P*(*Q*) is the self form factor which changes with the length and diameter of the particle. Information about the structure of scattering objects is usually obtained by comparing a scattering profile calculated from a presumed geometrical shape with the obtained data, and the process is iterated until an acceptable fit is produced. To enhance structural sensitivity and resolution, SANS measurements of protein structure are often done in D_2_O instead of H_2_O so that the interfacial boundary is highlighted. Although SANS is capable of detecting size and shape of scattering objects over a large size range, it is more sensitive to the small changes over range of a few nanometers compared to DLS.

**Figure 9:**
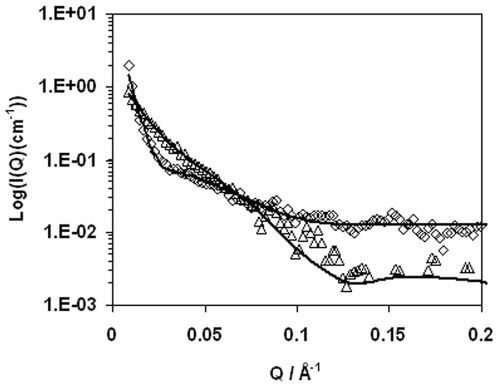
SANS scattering intensity profiles (I) plotted against wave vector (Q). The two sets of the measured data were from 2 mg/ml LipC in 20 mM Tris-HCl pH 8 (Δ); in 20 mM Tris-HCl pH 8 with 3.4 M NaCl at 45°C (◊). The continuous lines represented the best fitted curves, with the parameters obtained listed in Table 2. Error bars were not added for clarity.

In our previous studies, SANS was used to determine the solution structures of dimeric globular proteins of glucose oxidase and lactoferrin [37]. It was found that both molecules could be modeled into cylindrical shapes whose sizes matched their respective crystalline structures well. In this work, we have investigated the effects of pH, salt and temperature by undertaking selective SANS experiments with the fitted parameters listed in Table 2. The scattering profiles appeared to be similar in shape, indicating that the main features of solution characteristics were similar. Figure 9 compares the two representative profiles measured from the system at pH 8, 22°C without any added NaCl and from the same system with the addition of 3.4 M NaCl and at 45°C. Quantitative analysis was carried out by first assuming spherical shape for the hydrated enzyme. It was found that such a model could not produce any adequate fit over the size range appropriate for LipC. Subsequent attempts with cylindrical model improved the fits, but it was necessary to adopt at least two size distributions to account for polydispersity in each of the samples measured, consistent with the DLS analysis. In the absence of NaCl added, the model is sensitive to the diameters of the small cylinders, with an optimally fitted value of 6±0.5 nm. On the other hand, the fitting showed much less sensitivity to the length. Any values around 15 nm or greater could produce acceptable fits. As this length is clearly greater than the full length expected from the single enzyme molecule, the model implied the formation of small cylindrical aggregates, equivalent to the end-by-end templating between 2 or more enzymes. Larger cylindrical aggregates with a diameter of 20 nm and a length of 40 nm were also required for fitting the scattering curve, showing that under this solution condition, some of the enzyme molecules also formed aggregates through side-by-side and end-to-end templating. The scale factors produced from the fitting (Table 2) provided a useful indication of the population of both types of aggregates, with the small aggregates being dominant, consistent with DLS finding. Addition of 1.7 M NaCl did not alter the main feature of the picture, that is, the two types of scattering objects were still required to fit the measured scattering profile. The diameter of the cylinder remained 6±0.5 nm and the length of the small cylindrical aggregates found to be 20±5 nm. The scale factors again revealed that the proportion of the large aggregates was again low. When [NaCl] was increased to 3.4 M, the cylindrical diameter was still about 6 nm and other parameters remained largely the same. The scale factors also showed little change in the proportion of small to large aggregates. When the same measurement was made at 45°C, the small scattering objects had both diameter and length at 6 nm, consistent with the dimensions expected of unimers. However, the large aggregates became bigger. These SANS results thus showed that over the entire salt concentration range studied aggregates always existed and that as the salt concentration increased the size of the large aggregates increased but its proportion remained low, as indicated by the relative ratio of the scale factors. This trend of the effect of salt addition on the size of large aggregates is broadly consistent with the outcome of DLS results presented in Figure 8. More importantly, SANS studies also revealed that the coexistence of small cylindrical aggregates equivalent to the formation of dimers and that these became unimers at the optimal conditions. It is likely that these less aggregated enzymes were active than those heavily aggregated because of structural unfolding incurred.

**Table 2:** Best fit parameters obtained from analysis of SANS profiles.

pH	NaCl (M)	Temperature (°C)	Scale factor	Diameter (nm)	Length (nm)
8	0	22–23	7.2×10^−7^	6±0.5	≥15
			6.7×10^−8^	20	40
8	1.7	22–23	6.0×10^−7^	6±0.5	20±5
			2.2×10^−7^	40±4	12±2
8	3.4	22–23	5.3×10^−7^	6±0.5	20±5
			1.5×10^−7^	54±5	16±2
8	3.4	45	4.1×10^−7^	6±0.5	6±2
			1.2×10^−7^	>100	20±2
4	0	22–23	3.0×10^−7^	6±0.5	>20
			6.6×10^−7^	40±4	13±1

The scale factors provided a useful indication of proportion of two different scattering objects, unimers/dimers versus large aggregates, coexistent in a given solution. All the measurements were made at the LipC concentration of 2 mg/ml and 20 mM Tris-HCl buffer under different combinations of pH, temperature and [NaCl] as specified in the table.

The effect of pH was also examined by SANS. The parameters obtained from the analysis of the SANS scattering profile at pH 4 was also listed in Table 2. It was clear that at pH 4 the enzyme also existed in the form of mixed aggregates, with little indication of free molecules. The basic structural features were similar to those observed at pH 8, except the large aggregates became larger, consistent with the structural analysis from DLS (data not shown). Note that at pH 4 and 8 and the ambient temperature, the enzyme had little activity.

Thus, the SANS data as listed in Table 2 show that each solution studied could be approximated to a group of small aggregates of unimers and dimers coexistent with another group of large aggregates. Upon increase of NaCl concentration to around 3 M, the large aggregates became greater, indicating more extensive structural unfolding for some enzyme molecules, but the overall enzyme activity became substantially increased, showing that it was the unimers and dimers that might become active. Although DLS and SANS revealed the inhomogeneous nature of aggregation, it remains unclear why small aggregates (equivalent to dimers or trimers) formed at pH 4 and 8 had little activity. However, the CD spectra did reveal the large increase in the α-helical content with [NaCl] addition over the optimal activity range. Further CD work will be used to assess the combined temperature and salt effect on structure-function relation. It is intuitive to assume that the association of a large amount of hydrated amino acid residues in LipC with cations would substantially contribute to the stability and solubility of LipC, and the results presented in this work show that this is so as far as the average activity and secondary structure are concerned. But increase in [NaCl] also caused a substantial increase in enzyme aggregation. Hence, the overall picture was the formation of inhomogeneous solutions within which there were different responses to the increase of NaCl concentration.

In summary, we have cloned a halophilic esterase gene and expressed it in *E. coli*. The recombinant enzyme LipC was purified and characterized by combining both biochemical and biophysical measurements. MALDI-TOF peptide mass fingerprint spectral analysis of fragments of the halophilic esterase derived through trypsin digestion showed a complete matching to the sequence of the enzyme. The resultant enzyme showed an anomalous migration in SDS-PAGE, possibly due to its special amino acid composition. Preliminary characterization of enzymatic activity through the measurement of ester bond cleavage in *p*-nitrophenyl acylates indicated that its relative activity was dependent on salt concentration and temperature. The enzyme clearly showed preference to *p*-nitrophenyl esters with short acyl chains, consistent with the basic esterase character. Under the experimental conditions, the enzyme was found to show its optimal activity at pH 9.5, 45°C and [NaCl] = 3.4 M, or other equivalent conditions. However, an important criterion for enzymatic biocatalysis is its stability against time. Whilst salt addition could offer high enzymatic stability against time, this effect was found to be highly temperature dependent. As the reaction temperature increased, the salt stabilizing effect decreased. As the temperature was above 50°C, salt addition turned out to accelerate the destabilization.

The solution structure analysis of LipC by combining CD, DLS and SANS measurements led to interesting observations. The CD spectra revealed that under the optimal activity conditions the enzyme solutions showed the highest content of α-helical structure. The scattering data revealed that the highest enzyme activities matched the highest proportions of free molecules in solution. Given that even under the optimal activity conditions revealed, there were still some fractions of enzyme that existed in the form of aggregates, indicating that further effort is required to improve the solubility and stability of the enzyme in aqueous solution, particularly with respect to understanding the responses of the high content of acidic amino acids to temperature and salt under alkali pH.

As halophilic enzymes are becoming increasingly attractive, LipC is clearly a good model for further study of the structure-function relation, the striking salt tolerant ability and its structural implication on activity at different structural levels.

## Materials and Methods

### Cells, biochemicals and chemicals

*Escherichia coli* BL21 (DE3) and plasmid pET28a were obtained from Novagen (USA). Extaq polymerase was purchased from TaKaRa (Japan). Restriction enzymes were from Promega, and ultrapure deoxynucleotide solution (dNTPs) from Pharmacia Biotech (Sweden). Trypsin,Isopryl-β-D-thiogalactopyranoside (IPTG), *p*-nitrophenyl (PNP) esters (C2–C16, denoted as *p*-NP-Cn) and Fast Blue RR were from Sigma. All other chemicals were of the highest reagent grade commercially available.

### Gene cloning and sequencing

The gene lipC (Genbank accession number NC_006396) with lipase motif was chosen from the genome of Haloarcula marismortui ATCC 43049. Genomic DNA was extracted by Genome DNA Extraction Kit (Tiangen) according to the manufacture's instruction. The gene was amplified by PCR method using the following two primers with *Nhe*I and *Hin*dIII restriction sites: TATATAGCTAGCATGTCCACGACGG (upper primer, *Nhe*I cutting site as underlined); CGTCGCAAGCTTGAGCTACTTAAC (lower primer, *Hin*dIII cutting site as underlined). In order to allow easy purification the forward and reverse primers were designed to incorporate N-terminal as well as C-terminal 6×His affinity tags. The PCR was using 40 ng of Haloarcula marismortui ATCC 43049 genome DNA as template and the following parameters: initial denaturation (94°C, 4 min); followed by 30cycles of denaturation (94°C, 1 min), annealing (57°C, 2 min), and extension (72°C, 3 min) using ExtaqDNA polymerase. The purified PCR product purification was digested with *Nhe*I and *Hin*dIII restriction endonucleases (TaKaRa) and inserted in pET28a (Novagen Inc.) cut by the same endonucleases. The recombinant product was primarily transformed in maintenance host *E. coli* BL21 (DE3). DNA sequence result show the gene *lipC* was identical inserted in the plasmid.

### Recombinant protein expression and purification

*E. coli* BL21 (DE3) carrying plasmid pET28a-lipC was grown in LB medium. The expression of the recombinant protein was induced by adding IPTG (1 mM) after three hours of incubation at 37°C (to an OD_600_ of ∼0.6–0.8). Cells were harvested by centrifugation after overnight growth in the presence of IPTG at 28°C, and resuspended in 50 mM Tris-HCl buffer (pH 8.0). After ultrasonic disruption, the cell debris was removed by centrifugation (12000 rpm, 20 min, 4°C). The supernatant was applied to a Nickel affinity column (Ni-his NTA Novagen). The fraction containing esterase activity was collected and concentrated. After desalted the sample was applied to DEAE anion-exchange chromatography (Amersham Biosciences) and Superdex 200 10/300 GL gel filtration column (1.0×30 cm; Amersham Biosciences) for further purification. All steps were carried out according to the standard protocol at 4°C.

### Protein determination and electrophoreses

The protein concentration was measured using the Bio-Rad Protein Assay system, with BSA as a standard. SDS-PAGE was performed using 5% stacking gel and 10% resolving gel, and proteins were stained with Coomassie brilliant blue R-250. Esterase activity staining (zymogram) was performed after non-denaturing PAGE by incubating the gel in 100 ml Tris-HCl buffer (25 mM, pH 7.4) containing 50 mg α-naphthyl acetate, 30 mg Fast Blue RR Salt, 1 M NaCl and 1% (v/v) acetone.

### Enzyme assays

The esterase activity was measured by monitoring the hydrolysis of *p*-nitrophenyl acetate or other *p*-nitrophenyl acylates (Sigma) in 50 mM Tris-HCl buffer (pH 8.5). Each reaction mixture (2 ml) contained 0.5 mM substrate and any amount of NaCl (or KCl) as specified. The amount of *p*-nitrophenol liberated during the reaction was monitored continuously at 405 nm in a DU800 spectrophotometer (Beckman) with a temperature control module. One unit of activity was defined as the amount of enzyme that released 1 µmol of *p*-nitrophenol/min.

### MALDI-TOF mass spectrometry

The target band of LipC was collected from SDS-PAGE gel and placed in an Eppendorf tube for digestion. In-gel digestion was proceed with 0.01 mg trypsin (Sigma–Aldrich) over night at 37°C.1 µl of the digested peptide was spotted on a Anchorchip treated with matrix solution CHCA(Sigma) and washing with 0.1% TFA, then the crystallized sample was subjected to MALD-TOF-MS (ABI4700). MS analyse was carried out with standard method and the MS spectra was searched with the theoretical amino acid sequence of LipC.

### Circular dichroism (CD)

UV CD spectra between 205 and 250 nm were collected on a JASCO 720 spectropolarimeter using a 1 mm path length cuvette at the room temperature of 22–23 °C. Purified enzyme (0.4 mg/ml, pH 8) was measured in the presence of different concentrations of NaCl. Raw ellipticity data was converted to mean residue ellipticity before plotting.

### Dynamic light scattering (DLS)

DLS measures the intensity correlation function of light scattered from a sample solution. Analysis of the intensity correlation profiles provides the decay rate distribution from which the diffusion coefficient is determined. The Stokes-Einstein equation is then used to calculate the hydrodynamic radii of the protein. All DLS measurements were performed using a Malvern Instruments Nano-S Nanosizer. The instrument was fitted with a helium-neon laser (633 nm) and the detection angle was 173° with respect to the incoming beam. Samples were measured in a 1 cm path length quartz cell and the data were analyzed using Malvern Instruments Dispersion Technology Software. The refractive index for the protein was taken to be 1.45 with an absorbance of 0.001. The viscosity and refractive index of water were taken as 0.8872 cPa and 1.330, respectively. Three measurements were performed on each sample, with an average of three runs taken for each measurement.

### Small Angle Neutron Scattering (SANS)

The principle of SANS and the outline of data treatment have been described by King.[36] TrisHCl buffer (20 mM pH 8) in D_2_O was used as a solvent to provide the isotopic contrast. SANS experiments were carried out at LOQ, ISIS Neutron Facility, Rutherford Appleton Laboratory (RAL), Oxford, UK using neutron wavelengths ranging from 2.2 to 10 Å. The 64 cm square detector was at a distance of 4.1 m, giving a wave vector (Q) range from 0.006 to 0.28 Å^−1^. Samples were measured in 2.0 mm path length banjo silica cells. The data was fitted using Fish program provided by Dr Richard Heenan at RAL, Oxfordshire, UK.

## References

[1] Jaeger KE, Dijkstra BW, Reetz MT (1999). Bacterial biocatalysts: molecular biology, three-dimensional structures, and biotechnological applications of lipases.. Annu Rev Microbiol.

[2] Ollis DL, Cheah E, Cygler M, Dijkstra B, Frolow F (1992). The alpha/beta hydrolase fold.. Protein Engineering.

[3] Jaeger KE, Ransac S, Dijkstra BW, Colson C, Van Heuvel M (1994). Bacterial lipases.. FEMS Microbiol Rev.

[4] Reis P, Holmberg K, Watzke H, Leser ME, Miller R (2009). Lipases at interfaces: a review.. Advances in Colloid and Interface Science.

[5] Jaeger KE, Eggert T (2002). Lipases for biotechnology.. Current Opinion in Biotechnology.

[6] Panda T, Gowrishankar BS (2005). Production and applications of esterases.. Applied Microbiology and Biotechnology.

[7] Hasan F, Shah AA, Hameed A (2006). Industrial applications of microbial lipases.. Enzyme and Microbial Technology.

[8] Herbert RA (1992). A perspective on the biotechnological potential of extremophiles.. Trends in Biotechnology.

[9] Van den Burg B (2003). Extremophiles as a source for novel enzymes.. Curr Opin Microbiol.

[10] Cho AR, Yoo SK, Kim EJ (2000). Cloning, sequencing and expression in Escherichia coli of a thermophilic lipase from Bacillus thermoleovorans ID-1.. FEMS Microbiology Letters.

[11] Demirjian DC, Moris-Varas F, Cassidy CS (2001). Enzymes from extremophiles.. Curr Opin Chem Biol.

[12] Imamura S, Kitaura S (2000). Purification and characterization of a monoacylglycerol lipase from the moderately thermophilic Bacillus sp. H-257.. Journal of Biochemistry (Tokyo).

[13] Li H, Zhang X (2005). Characterization of thermostable lipase from thermophilic Geobacillus sp. TW1.. Protein Expr Purif.

[14] Schmidt-Dannert C, Rua ML, Atomi H, Schmid RD (1996). Thermoalkalophilic lipase of Bacillus thermocatenulatus. I. molecular cloning, nucleotide sequence, purification and some properties.. Biochimica et Biophysica Acta.

[15] Kojima Y, Yokoe M, Mase T (1994). Purification and characterization of an alkaline lipase from Pseudomonas fluorescens AK102.. Bioscience, Biotechnology, and Biochemistry.

[16] Jinwal UK, Roy U, Chowdhury AR, Bhaduri AP, Roy PK (2003). Purification and characterization of an alkaline lipase from a newly isolated Pseudomonas mendocina PK-12CS and chemoselective hydrolysis of fatty acid ester.. Bioorg Med Chem.

[17] Breuil C, Kushner DJ (1975). Lipase and esterase formation by psychrophilic and mesophilic Acinetobacter species.. Canadian Journal of Microbiology.

[18] Pratuangdejkul J, Dharmsthiti S (2000). Purification and characterization of lipase from psychrophilic Acinetobacter calcoaceticus LP009.. Microbiol Res.

[19] Luo Y, Zheng Y, Jiang Z, Ma Y, Wei D (2006). A novel psychrophilic lipase from Pseudomonas fluorescens with unique property in chiral resolution and biodiesel production via transesterification.. Applied Microbiology and Biotechnology.

[20] Gonzalez C, Gutierrez C (1970). Presence of lipase among species of extremely halophilic bacteria.. Canadian Journal of Microbiology.

[21] Oren A, Ginzburg M, Ginzburg BZ, Hochstein LI, Volcani BE (1990). Haloarcula marismortui (Volcani) sp. nov., nom. rev., an extremely halophilic bacterium from the Dead Sea.. International Journal of Systematic Bacteriology.

[22] Boutaiba S, Bhatnagar T, Hacene H, Mitchell DA, Baratti JC (2006). Preliminary characterisation of a lipolytic activity from an extremely halophilic archaeon, Natronococcus sp.. Journal of Molecular Catalysis B: Enzymatic.

[23] Amoozegar MA, Salehghamari E, Khajeh K, Kabiri M, Naddaf S (2008). Production of an extracellular thermohalophilic lipase from a moderately halophilic bacterium, Salinivibrio sp. strain SA-2.. Journal of Basic Microbiology.

[24] Kim J, Dordick JS (1997). Unusual salt and solvent dependence of a protease from an extreme halophile.. Biotechnology and Bioengineering.

[25] Madern D, Ebel C, Zaccai G (2000). Halophilic adaptation of enzymes.. Extremophiles.

[26] Norberg P, von Hofsten B (1969). Proteolytic enzymes from extremely halophilic bacteria.. Journal of General Microbiology.

[27] Bhatnagar T, Boutaiba S, Hacene H, Cayol JL, Fardeau ML (2005). Lipolytic activity from Halobacteria: screening and hydrolase production.. FEMS Microbiol Lett.

[28] Sana B, Ghosh D, Saha M, Mukherjee J (2007). Purification and characterization of an extremely dimethylsulfoxide tolerant esterase from a salt-tolerant Bacillus species isolated from the marine environment of the Sundarbans.. Process Biochemistry.

[29] Baliga NS, Bonneau R, Facciotti MT, Pan M, Glusman G (2004). Genome sequence of Haloarcula marismortui: a halophilic archaeon from the Dead Sea.. Genome Res.

[30] Müller-Santos M, Souza EMd, Pedrosa FdO, Mitchell DA, Longhi S (2009). First evidence for the salt-dependent folding and activity of an esterase from the halophilic archaeon Haloarcula marismortui.. Biochimica et biophysica acta.

[31] Arpigny JL, Jaeger KE (1999). Bacterial lipolytic enzymes: classification and properties.. Biochem J.

[32] Monstadt GM, Holldorf AW (1991). Arginine deiminase from Halobacterium salinarium. Purification and properties.. Biochemical Journal.

[33] Toniolo C, Bonora GM, Salardi S, Mutter M (1979). Linear Oligopeptides. 57. A Circular Dichroism Study of α-Helix and β-Structure Formation in Solution by Homooligo-L-methionines.. Macromolecules.

[34] Follmer C, Pereira FV, Silveira NPd, Carlini CR (2004). Jack bean urease (EC 3.5.1.5) aggregation monitored by dynamic and static light scattering.. Biophy Chem.

[35] Svergun DI, Richard S, Koch MHJ, Sayers Z, Kuprin S (1998). Protein hydration in solution: Experimental observation by x-ray and neutron scattering.. Proc Natl Acad Sci USA.

[36] King SM, Dawkins RAPJV (1999). Small Angle Neutron Scattering.. Modern Techniques for Polymer Characterisation.

[37] Lu JR, Perumal S, Zhao X, Miano F, Enea V (2005). Surface-Induced Unfolding of Human Lactoferrin.. Langmuir.

